# Measurement of Ultrafine Particles and Other Air Pollutants Emitted by Cooking Activities

**DOI:** 10.3390/ijerph7041744

**Published:** 2010-04-16

**Authors:** Qunfang Zhang, Roja H. Gangupomu, David Ramirez, Yifang Zhu

**Affiliations:** 1 Texas A&M University-Kingsville, Department of Environmental Engineering, 700 University Blvd. MSC 213, Kingsville, TX 78363, USA; E-mails: zhangqunfang@gmail.com (Q.Z.); kfdr000@tamuk.edu (D.R.); 2 The University of Texas at Arlington, Department of Civil and Environmental Engineering, Box 19308 416 Yates St. Suite 425, Arlington, TX 76019, USA; E-mail: rojaharitha@yahoo.com

**Keywords:** cooking emissions, cooking style, cooking parameters, spatial profile

## Abstract

Cooking emissions show a strong dependence on cooking styles and parameters. Measurements of the average ultrafine particle (UFP) concentration, PM_2.5_ and black carbon concentrations emitted by cooking activities ranged from 1.34 × 10^4^ to 6.04 × 10^5^ particles/cm^3^, 10.0 to 230.9 μg/m^3^ and 0.1 to 0.8 μg/m^3^, respectively. Lower UFP concentrations were observed during boiling, while higher levels were emitted during frying. The highest UFP concentrations were observed when using a gas stove at high temperature with the kitchen exhaust fan turned off. The observed UFP profiles were similar in the kitchen and in another room, with a lag of approximately 10 min.

## 1. Introduction

Americans typically spend the majority of their time indoors, making exposure to indoor air pollution a significant health concern. Indoor pollutant sources include smoking, cleaning, consumer products (*i.e.*, paints and deodorizers) and cooking activities [1,2]. Besides smoking, cooking has been identified as another major source of indoor air pollution. Liao *et al.* [3] showed that Chinese-style cooking contributed approximately 30% to indoor concentration of particles from 0.5 to 5 μm. Cooking activities can emit gaseous pollutants and particulate matter (PM), both of which have impacts on health. For example, polycyclic aromatic hydrocarbons (PAHs) and aldehydes emitted from cooking activities were shown to have potential carcinogenic effects on both humans and animals [4–7]. PM emitted from cooking oil fume has been associated with respiratory problems, lung cancer and cardiopulmonary deaths [8,9]. Ultrafine particles (UFPs, diameter < 100 nm) are major components of PM on a number basis. On an equal mass basis, UFPs have been shown to be more toxic than larger particles to laboratory animals and humans due to the smaller size and larger surface area of these particles [10–14].

High emissions of PM from cooking activities have been reported in many previous studies. Wallace *et al.* [15] determined that cooking was associated with both an increase of a factor of 10 in the concentration of UFPs and an increase of a factor of 3 in PM_2.5_. Similarly, Li *et al.* [16] observed a 10-fold increase in submicron particles when frying chicken on gas stoves. He *et al.* [17] reported that grilling Chinese-style food led to elevated submicron particle and PM_2.5_ concentrations that were up to 5 and 90 times higher than normal, respectively.

Cooking emissions depend strongly on a variety of parameters, including ingredients, type of stove and cooking temperature. Large variations in cooking-based PM concentrations have been observed under different conditions. Lee *et al.* [18] measured the indoor air quality in a Korean barbecue restaurant, a Chinese hot pot restaurant and a Chinese dim sum restaurant. The results showed that the highest PM exposure occurred in the Korean barbecue restaurant due to the use of pan-frying, while the lowest occurred in the Chinese dim sum restaurant, which mostly used steaming. Cooking emissions from 5 different cooking methods—steaming, boiling, stir-frying, pan-frying and deep-frying—were further investigated in a domestic kitchen [19]. The results showed that deep-frying generated the most PM_2.5_, while steaming and boiling generated the least. Buonanno *et al.* [20] conducted a study to characterize particle emissions during grilling and frying as a function of the type of food, source, cooking temperature and type of oil. Higher emission factors were reported at higher cooking temperatures and with fattier foods. Particle emission factors also varied significantly as a function of the type of oil used.

Although previous studies have provided substantial data about cooking emissions, the parameters that influence these emissions remain unclear. Cooking style varies with population, culture, climate and geographical location, which complicates the human risk assessment on cooking emissions. Different cooking styles employ different ingredients, cooking procedures and temperatures. How these factors affect particle generation and transport are still poorly understood. Thus, it is necessary to study cooking emissions from various cooking styles. In addition, few studies have addressed the impacts of exhaust fan setting, an important factor affecting exposures.

This study assesses the effects of a range of cooking styles and parameters on UFPs, PM_2.5_ and black carbon (BC) emissions in two stages. First, Indian, Chinese and Italian cooking styles were used at high temperature on an electric stove with the exhaust fan turned on. Subsequently, a single representative cooking activity (frying chicken) was used both to eliminate the variability associated with different cooking styles and to focus on the impacts of stove type, cooking temperature and exhaust fan setting.

## 2. Method

### 2.1. Sampling Site

The first stage of the study was conducted in a one–story, 140-m^2^ single family house (R1) with three bedrooms, one study room, one living room and one kitchen. R1 had a central air conditioning unit that served the entire house. The kitchen had a ceramic tile floor. The living room had hardwood floors partially covered with rugs, and the bedrooms and study room were carpeted. The kitchen had an electric stove with a recirculating exhaust fan that pulled air on the top of the stove through a filter, and blew it back to the kitchen. The monitoring instruments were placed 1 m from the stove with a sampling inlet facing the stove at the height at which an inhabitant would breathe in cooking emissions. Total particle number concentration, size distribution of UFPs, and PM_2.5_ mass concentration in the study room, located about 8 m from the kitchen, were simultaneously monitored to assess the spatial dispersion of the emissions. The study room was directly connected to the living room. During the measurements, central air conditioning systems were turned on. The doors and the windows were closed. The CO_2_ decay method reported by He *et al.* [17] was employed to determine the air exchange rate (AER). The AERs were found to be 0.45 h^−1^ (SD *=* 0.03 h^−1^) in this residence.

The second stage of the study focused on the effects of stove type, cooking temperatures and kitchen exhaust fan, and it was conducted in a student dorm (S1) and in two two-bedroom apartments (A1 and A2). The student dorm was approximately 20 m^2^ in size with ceramic tile floor throughout. Both apartments were approximately 60 m^2^ in size with ceramic tile floor in the kitchen and carpet in the living room and bedrooms. S1 and A1 had an electrical stove and A2 had a gas stove. Each studied site had a recirculating exhaust fan in the kitchens. Central air conditioning systems were turned on with the doors and the windows closed in all three residences during the measurements. The AERs were 0.39 h^−1^ (SD *=* 0.02 h^−1^) in S1, 0.28 h^−1^ (SD *=* 0.05 h^−1^) in A1 and 0.31 h^−1^ (SD *=* 0.04 h^−1^) in A2.

### 2.2. Instrument

The pollutants monitored in the kitchens included size distribution of UFPs, total particle number concentration, PM_2.5_ mass concentration and BC mass concentration. A scanning mobility particle sizer (SMPS; 3936L85, TSI Inc.) was used to measure the size distribution of particles with diameter in the range of 7.6 to 289 nm. The instrument consists of two components: (1) a Model 3080 Electrostatic Classifier with a Model 3081 Long Differential Mobility Analyzer used to select particles of a given size and (2) a water-based condensation particle counter (CPC; 3785, TSI Inc.). A second water-based CPC (3785, TSI Inc.) was used to measure the total number concentration of particles with size ranging from 5 to 6 nm to a few microns.

A TSI DustTrak photometer (Model 8520 TSI, Inc.) with a PM_2.5_ inlet impactor was used to continuously monitor particle mass concentration. The DustTrak was calibrated against a TEOM^®^ (Series 1400A, Thermo Scientific Co.) that measures gravimetric PM_2.5_ and PM_10_ mass concentrations at an air monitoring station maintained by the Texas Commission on Environmental Quality. A difference of a factor of 2.4 was found between the two instruments, and it was used to correct the DustTrak measurements. This correction factor was similar to that found by Yanosky *et al.* [21].

An aethalometer (AE-42-2, Magee Scientific, Inc.) was used to measure the elemental carbon concentration in near-real time. A sample cycle of 1 min was used. Data were continuously logged into an internal data logger. The factory calibration was used for this instrument.

To determine AERs, CO_2_ concentrations were measured by a TSI Q-trak indoor air quality monitor (Model 8550, TSI Inc., St. Paul, MN) at 30-s intervals. Q-trak was calibrated by calibration gas (1000 ppm CO_2_ and 35 ppm CO) and a wet and dry bulb thermometer.

In the study room in R1, a SMPS, a CPC and a DustTrak were used to simultaneously collect pollutant concentrations. These instruments were the same models as those used in the kitchen with one exception. The SMPS used in the study room consists of a Model 3080 Electrostatic Classifier with a Model 3085 Nano Differential Mobility Analyzer which selects particles in the range of 2.5 to 79.1 nm.

### 2.3. Sampling Protocol

Before each cooking episode, background concentrations of UFPs, PM_2.5_ and BC were measured for 15 min. The cooking process was then initiated by simultaneously turning on the stove and, where applicable, the exhaust ventilation. Both stove and exhaust ventilation were turned off once the cooking was complete. UFPs and other air pollutants were continuously measured until the particle number concentrations returned to background levels.

For the first phase of the study, the effect of cooking style on emissions was assessed using Indian, Chinese and Italian cooking styles. Indian cooking involved pan-frying chicken, peppers and vegetables. Chinese cooking involved frying chicken, shrimp and vegetables in a wok. Italian cooking involved boiling pasta and subsequently stir-frying it with vegetables. Each cooking experiment resulted in 5 to 6 servings. Cooking time ranged from 0.5 to 1 hour, depending on the dish being prepared. The electric stove was turned on with the dial at full power, and the exhaust fan was turned on for each cooking activity.

For the second part of the study, the effects of cooking conditions were assessed while frying chicken. Frying chicken was chosen as a representative cooking activity, and it was used in all experiments to exclude the variability in emissions caused by differences in cooking styles. A pan was heated for 1 min, after which 50 mL of corn oil were poured into it. Then 1.5 lb of seasoned chicken breast were added and slowly stirred until browning was observed. Stove type, cooking temperature and ventilation settings were varied to assess the factors affecting cooking emissions and exposures. Each factor had two settings: electricity or gas for stove power, high or medium for temperature and on or off for exhaust fan (Table 1). “High” and “medium” temperatures refer to setting the dial to full and medium power, respectively.

## 3. Results and Discussion

### 3.1. Characteristics of Cooking Emissions

**Figure 1.**
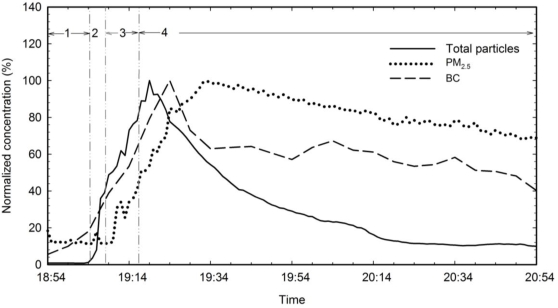
Normalized concentrations of total particle number concentration, PM_2.5_ mass concentration and BC mass concentration measured while cooking at high temperature on an electric stove with the exhaust fan turned on. The episode consisted of 4 periods: (1) background testing, (2) heating the pan and the oil, (3) frying the chicken and (4) post-cooking decay of emissions. Normalized concentrations are the fraction of pollutant concentrations based on the maximum concentrations measured in this cooking episode as follows: total particle number concentration: 4.27 **×** 10^5^ particles/cm^3^, PM_2.5_: 42.2 μg/m^3^ and BC: 0.6 μg/m^3^.

Figure 1 shows typical time series of total particle number, PM_2.5_ mass and BC mass concentrations emitted during high-temperature cooking using an electric stove with the exhaust fan turned off. The cooking episode was divided into 4 periods: (1) background testing, (2) heating of the pan and the oil, (3) frying the chicken and (4) post-cooking decay of emitted particles. The average background number concentration of all particles was 3.72 × 10^3^ particles/cm^3^. It increased rapidly when heating the oil and up to 3.64 × 10^5^ particles/cm^3^ after frying the chicken. The total particle number concentrations continued to increase for 5 min after the stove was turned off. Similar trends were observed for PM_2.5_ and BC mass concentrations. The PM_2.5_ mass concentration increased from a background concentration of 5.0 μg/m^3^ to a maximum of 42.2 μg/m^3^ measured 15 min after the stove was turned off. BC mass concentrations reached 0.6 μg/m^3^ 7 min after the stove was turned off.

Table 1 summarizes the operational conditions and the average concentrations of air pollutants for each cooking episode, which was from when the stove was turned on to 30 min after the stove was turned off. UFP concentration was defined as the sum of the number concentrations of particles with size between 7.6 and 289 nm, as measured by the SMPS. The average concentrations ranged from 1.34 × 10^4^ particles/cm^3^ to 6.04 × 10^5^ particles/cm^3^ for UFP, 10.0 μg/m^3^ to 230.9 μg/m^3^ for PM_2.5_ and 0.1 μg/m^3^ to 0.8 μg/m^3^ for BC. Large variations in all three parameters were observed for different cooking styles. The lowest average UFP concentrations were found when pasta and salad were prepared, while the highest were measured while frying chicken. Boiling pasta and stirring salad were water-based cooking methods and frying chicken was oil-based. Water-based cooking requires much less oil than oil-based one. The observed difference on UFP concentrations may be attributed to the different usage of oil. Buonanno *et al.* [20] reported significant higher emission factors when cooking foods containing a high percentage of fat than low fat vegetables.

Based on SMPS reported UFP number based size distribution data, surface area and mass concentration were calculated and averaged over all 14 cooking activities. The average number-based UFP distribution exhibited a mode around 70 (GSD = 10) nm. This value is in agreement with that measured in a previous study that found frying produced peak number concentrations of UFPs at about 60 nm, with a secondary peak at 10 nm [15]. The average surface area- and mass-based modes were 120 nm (GSD = 22 nm) and 160 nm (GSD = 31 nm), respectively.

### 3.2. Factors Affecting Cooking Emissions and Pollutant Decay

**Figure 2.**
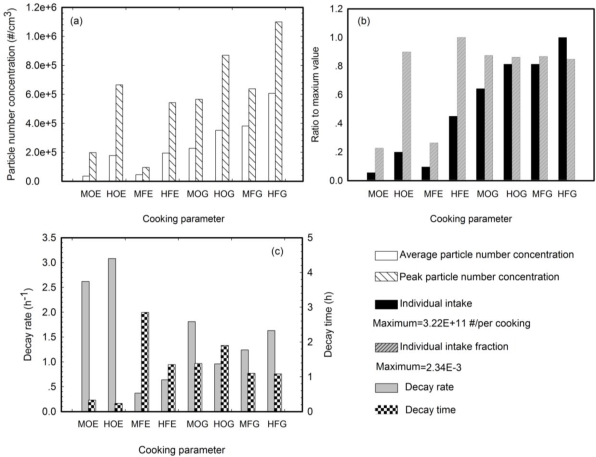
Effects of cooking parameters on (a) particle emission, (b) individual intake and intake fraction and (c) decay of total particle number concentration. “E” and “G” denote electric stove and gas stove; “M” and “H” denote medium and high temperature; and “O” and “F” refer to ventilation on and ventilation off, respectively.

Stove type and cooking temperature affected cooking emissions and exhaust fan setting affected pollutant decay. Figure 2 demonstrates the effect of these factors on UFP emissions, expected pollutant intake by an individual and the decay rate of total particle number concentrations. In this figure, the abscissa gives the operational conditions: “E” or “G” denotes electric or gas stove; “M” or “H” denotes medium or high temperature; and “O” or “F” denotes on or off for the ventilation. Figure 2(a) shows that the gas stove generated more particles than the electric stove, regardless of the cooking temperature and the exhaust fan setting. The kitchen ventilation system removed 41% of the total particles when using the gas stove and 16% when using the electric stove. High temperatures generated between 55% and 400% more particles than medium temperatures. The maximum particle number concentration was most strongly influenced by temperature. Even though the average total particle number concentrations measured for “EHO” and “EHF” conditions were much lower than those observed for “GHO” and “GHF” conditions, the peak particle number concentrations were comparable for the two types of stoves.

Measurements of air pollutant concentrations in a microenvironment alone are sometimes insufficient for assessing the associated adverse health risk. A more useful epidemiological parameter is the human intake of air pollutants from a given source. Individual intake is defined as the pollutant inhaled by an individual [22]. For cooking activities, individual intake of particles can be calculated as:

(1) $$\text{Individual}\;\text{intake}=\int_{0}^{Te}C(t)Rdt$$

where C(t) is the total particle number concentration in the kitchen at the time *t* (particles/cm^3^), *R* is the breath rate for an adult (cm^3^/min) and *Te* is the exposure time (min). We used an average respiratory rate of 0.5 m^3^/h as given by Adams *et al.* [23]. Exposure time was defined as the period from when the stove was turned on to 30 min after the stove was turned off, including both cooking and meal time. To more directly link a given emission source to its corresponding human intake, the intake fraction can be defined. Individual intake fraction is the ratio of the individual intake (Equation 1) to the total emissions from the source [22,24]. It is calculated as:

(2) $$\text{Individual}\;\text{intake}\;\text{fraction}=\frac{\text{Individual}\;\text{intake}}{ET_{c}}=\frac{\int_{0}^{Te}C(t)Rdt}{ET_{c}}$$

where *E* is the emission factor (EF) of each cooking activity (particles/min) and *Tc* is the cooking time (min). EF was calculated using a simplified equation reported by He *et al.* [17]. In our study, the EFs ranged from 1.23 × 10^12^ to 1.31 × 10^13^ particles/min, at the same order as the EFs in He *et al.* [17] and Buonanno *et al.* [20]. The health risk associated with cooking can thus be estimated by multiplying the total cooking emissions by the intake fraction and then by a health risk factor [25]. The result is a simplified measure that can be used for environmental health risk assessment.

Human exposure to cooking emissions is shown in Figure 2(b). Individual UFP intake ranged from 1.80 × 10^10^ to 3.22 × 10^11^ UFPs per cooking episode. The greatest individual intake was observed over the gas stove when cooking at high temperature with the exhaust fan turned off, and the lowest was measured over the electric stove at medium temperature with the exhaust fan on. The individual intake fractions ranged from 5.47 × 10^−4^ to 2.34 × 10^−3^ and were consistent in magnitude with the individual environmental tobacco smoke fraction (1.40 × 10^−3^) reported by Klepeis [26]. For the gas stove, the individual intake fractions were at similar level under different conditions. However a great difference was observed for the electric stove due to cooking temperature. At high temperature, the individual intake fraction for the electric stove was close to that for the gas stove, while at medium temperature it decreased by 75%.

Figure 2(c) shows the rate of total particle number concentration decay and the time it took for the concentration to decrease to background level. The time series of total particle number concentration after the stove was turned off was fitted into a natural exponential decay curve (Equation 3),

(3) $$C(t)=C_{0}\text{exp}(-kt)$$

where *C_0_* and *C(t)* are the number concentrations of total particle when the stove was turn off and at *t* h after it was off. The decay rate was determined by the coefficient part (*k*) of the exponent. The correlation coefficient *r^2^* was usually higher than 0.90. For the electric stove, the exhaust fan greatly accelerated the particle decay. At both temperatures, the ventilation-on condition achieved a decay rate that was 5 times faster than that found when ventilation was off. For the gas stove, the impact of the exhaust fan setting was not as notable. The extremely high concentration of particles from the gas stove limited the efficiency with which the exhaust fan could remove particles by filtration. Temperature had a smaller effect than ventilation on the decay rate. The decay rate was between 18% and 73% higher for high temperature cooking than for medium temperature cooking. This difference can be explained by the occurrence of coagulation and deposition when the total particle number concentration was high.

**Figure 3.**
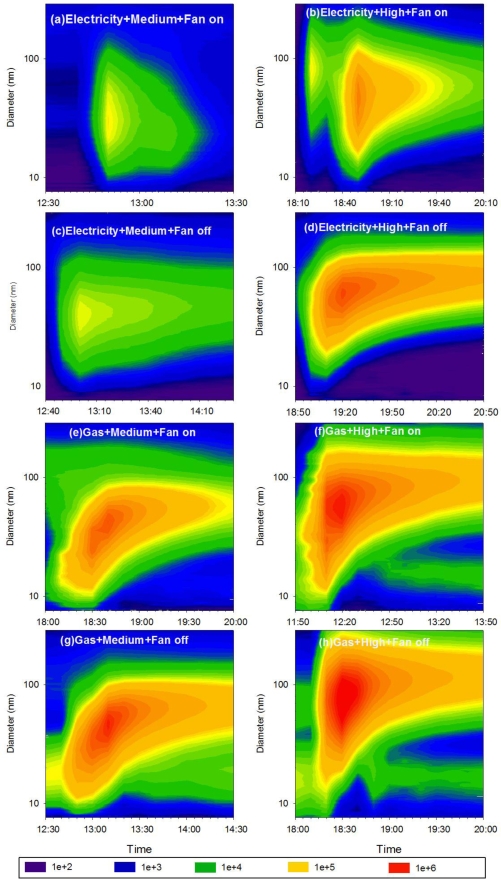
Number-based UFP size distributions as a function of time, shown for each set of cooking condition.

Contour plots of number-based UFP size distribution for each cooking activity are shown in Figure 3. The abscissa denotes the time when the data were collected, and the ordinate gives the particle size on a logarithmic scale. The color intensity indicates the normalized particle number concentration (dN/dLogDp) for a given particle size at a given time. The gas stove emitted higher UFP concentrations than the electric stove, and UFP number concentrations were higher at high temperature than at medium temperature. Emitted UFPs were characterized by a unimodal distribution. The mode size of UFPs at medium temperature ranged from 30 to 50 nm, smaller than the high temperature mode size of 60 to 90 nm. A shift in intensity (orange and red area) from small size to large size with time is evident in Figure 3 for all scenarios except the electric stove at medium temperature, which had a relatively low concentration of UFPs. This shift indicates that coagulation occurred when emitted particles reached high concentrations.

**Figure 4.**
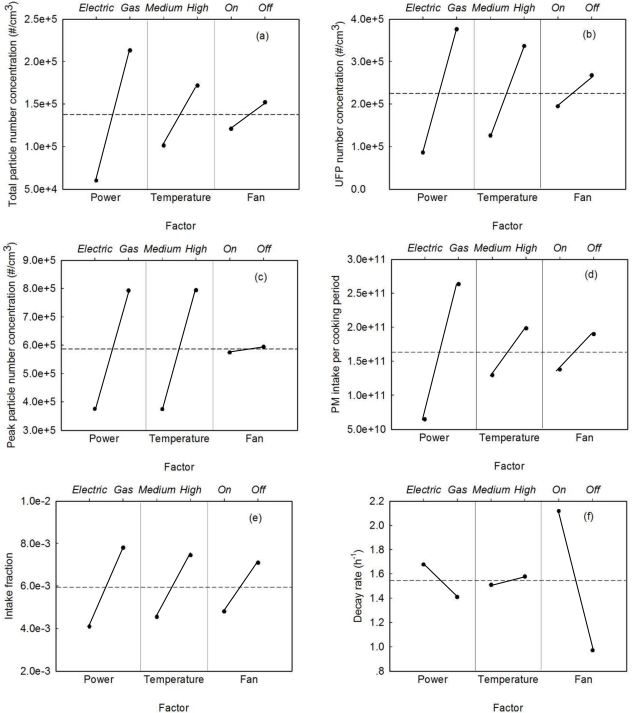
Results of factor analysis showing the effect of stove type, exhaust fan setting and temperature on (a) average total particle number concentration, (b) average UFP number concentration, (c) peak particle number concentration, (d) PM intake per cooking period, (e) intake fraction and (f) decay rate.

A three-factor two-level factorial analysis was applied to quantify the effect of the analyzed variables (stove type, cooking temperature and exhaust fan) on the output parameters (average total particle number concentration, UFP number concentration, peak total particle number concentration, PM intake, intake fraction and decay rate). The main impacts of the three variables are shown in Figure 4. The lower abscissa gives the variables, the upper abscissa gives the two options for each variable, and the ordinate denotes the output parameters. The dashed line indicates the mean value of each parameter and the solid black dots give the value of the outputs for each setting of each variable. The slope of the line between the two dots was used to analyze the significance of the factor, with a larger slope corresponding to a more significant factor.

The type of stove had the most significant effect on all output parameters analyzed here except decay rate. The decay rate was largely determined by the exhaust fan setting. As shown in Figure 4(f), turning on the fan increased the decay rate by a factor of 2. The exhaust fan had a moderate impact on all other parameters except peak particle number concentration. The decay rate reflects the removal rate of particles and was enhanced by ventilation, which is one mechanism for particle removal. The other parameters, such as average particle number concentration, reflect competition between particle generation and particle removal, explaining the smaller impact of ventilation on these parameters. The peak particle number concentration was primarily determined by factors related to particle generation such as stove type and temperature (see Figure 4(c)). Temperature did not affect the decay rate but had a remarkable impact on particle emissions.

### 3.3. Spatial Dispersion of Cooking Emissions

**Figure 5.**
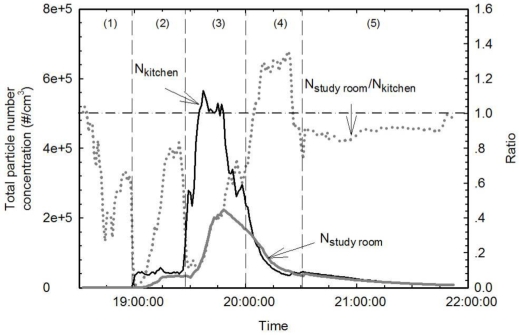
Time series of total particle number concentration emitted while cooking on an electric stove at high temperature with the exhaust fan turned on. Concentrations are shown for both the kitchen and the study room. The gray dotted line indicates the ratio of the total particle number concentration in the kitchen to that in the study room.

Figure 5 shows the time series of total particle number concentration resulting from Indian-style cooking of onions, green peppers and chicken. Results are shown for the kitchen (K) as a black solid line and for the study room (S) as a gray solid line. The S/K ratio is shown as a dotted gray line. The kitchen was not isolated from the rest of the house. Air pollutants emitted from cooking activities could be dispersed to other rooms in the same residence where some susceptible population might stay such as children and senior citizens. Therefore the health risk from cooking emissions may be underestimated if human exposure is only considered in the kitchen. The S/K ratio provides important information on the spatial distribution of air pollutants from cooking activities and may facilitate future health risk assessment on cooking emissions. The cooking activity was divided into 5 steps: (1) background testing, (2) turning on the ventilation, heating the pan and stir-frying the onion with oil at medium temperature, (3) stir-frying the peppers and chicken at high temperature, (4) turning off the stove while keeping the ventilation on and (5) turning off the ventilation.

At the beginning of Step 1, the S/K ratio was around 1 and showed a steady decrease before cooking began. The total particle number concentration in the kitchen was slightly higher than that in the study room during Step 2 due to preparatory activities such as washing, cutting and walking in the kitchen. Once the stove was turned on, the total particle number concentration in the kitchen increased rapidly from 1.02 × 10^3^ particles/cm^3^ to 4.28 × 10^4^ particles/cm^3^. Such a rapid increase was not observed in the study room. About 5 min later, the total particle number concentration in the study room began to increase slowly and reached 3.59 × 10^4^ particles/cm^3^. The total particle number concentration kept increasing while the peppers and chicken were cooked. Total concentration eventually reached a peak of 5.66 × 10^5^ particles/cm^3^ in the kitchen, 550 times higher than background concentrations. The highest total particle number concentration in the study room was 2.24 × 10^5^ particles/cm^3^, and it was measured 12 min after the peak was reached in the kitchen. The S/K ratio varied between 0.59 and 0.65 during the last 10 min of Step 3. When the stove was turned off, ventilation from the exhaust fan enhanced the decay rate in the kitchen, where concentration decayed 46% faster than in the study room. As a result, the S/K ratio increased to 1.3. When the exhaust fan was turned off, the decay rates in the kitchen and in the study were both converged to 1.3 h^−1^.

**Figure 6.**
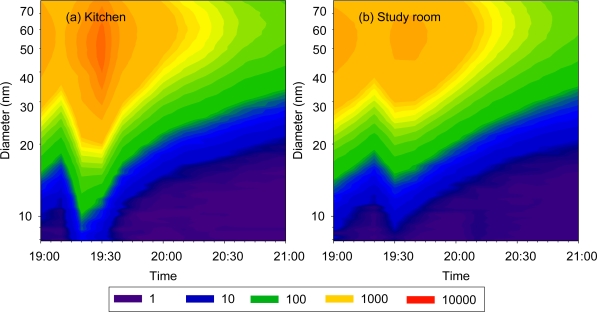
Contour plots of ultrafine particle number size distribution during a cooking episode in (a) the kitchen and (b) the study room.

The UFP size distributions in the kitchen and the study room are compared in Figure 6. The size distributions in both rooms were unimodal with a primary mode of 60 to 70 nm. The UFP concentrations were lower in the study room than in the kitchen with a lag time of about 10 min.

### 3.4. Health Risk Implication and Limitation

Results from this study showed people were exposed to high levels of UFPs, up to 550 times more than background during cooking time. UFPs are of serious health concerns because of their small size, large surface area, and toxic pollutants such as PAHs absorbed on these particles. Significant percentage of Asian non-smoking women were found with lung cancer [27,28], which may attribute to long term exposure to cooking fume. The human health risk assessment on UFPs emitted by cooking was reported by See *et al.* [29]. The levels of non-carcinogenic and carcinogenic risk were 50% and 111 times higher than the acceptable levels, respectively.

The exposure to UFPs from cooking activities was not confined to kitchen. The measurement in the study room revealed that with an open kitchen, UFPs were easily dispersed to other rooms in the occupied residence. Even thought the concentration was lower than that in the kitchen, it was still up to 270 times higher than non-cooking time. Since susceptible population such as children and elderly may stay in these rooms during cooking time, risk assessment on cooking emissions need to include all residences. Studies on the spatial distribution of cooking emissions in an occupied residence may provide useful information for future epidemiological study design.

This study also showed great variability on air pollutant concentrations emitted from cooking of different styles and under different conditions. This indicates health risk of cooking emissions should be evaluated on a case-by-case basis. The factors, such as diet habit and energy supply, should be taken into consideration. Moreover, the public should be informed that some simple methods can be taken to significantly reduce exposure to UFPs from cooking. These methods include using electric stoves instead of gas stoves, avoiding to cook at high temperature, keeping exhaust fan on during cooking, and if possible, separating the kitchen from other rooms by closing doors or installing a high efficient ventilation device in the kitchen.

It is noted only 4 cooking styles were studied and the measurements were conducted at four sites. The styles employed in our study did not cover all dishes, thus the results are only applicable to the population using these studied styles. Since our study was done in real residences which seldom had both gas and electric stoves, the sampling sites might have potential influence on UFP transport and transformation due to building geometry, ventilation system and building material. Further studies with additional repeated measurements may advance the knowledge on cooking emissions and achieve statistically solid conclusions.

## 4. Conclusions

Cooking was found to be a significant indoor source of UFPs. Cooking increased the UFP concentrations in the kitchen by up to a factor of 550. The average UFP number concentration, PM_2.5_ mass concentration and BC mass concentration ranged from 1.34 × 10^4^ to 6.04 × 10^5^ particles/cm^3^, 10.0 to 230.9 μg/m^3^ and 0.1 to 0.8 μg/m^3^, respectively.

Cooking emissions varied greatly depending on the cooking styles and parameters used. The lowest average UFP concentrations were observed during boiling, while the highest were measured during frying. The highest average UFP concentrations were observed during high-temperature cooking on a gas stove with the kitchen exhaust fan turned off. When using an electric stove at medium temperature with the exhaust fan turned on, the average UFP concentration was reduced to only 5% of the maximum. Stove type had the most significant effect on all the variables analyzed here. Temperature also played a significant role in driving particle emissions and intakes, leading to a particularly strong impact on peak particle number concentrations. The exhaust fan had the most influence on the decay rate. Turning on the fan increased the decay rate by a factor of 2.

The total particle number concentrations in the study room were comparable to those in the kitchen with a lag of 10 to 12 min. The size distributions in both rooms were similar, with a primary mode of about 60 to 70 nm. However, the peak concentration in the study room, 2.24 × 10^5^ particles/cm^3^, was only 40% of that in the kitchen.

## Figures and Tables

**Table 1.** Summary of average concentrations during cooking activities.

No.	Experimental Variables							Environmental parameters		
	Site	Style	Ingredients	Stove	Fan	Temp.	Cooking time (min)	UFPs (×10^5^ #/cm^3^)	PM_2.5_ (μg/m^3^)	BC (μg/m^3^)
1	R1	Indian	Chicken & rice	E	On	H	62	1.13	94.3	0.6
2	R1	Indian	Egg & vegetable	E	On	H	36	0.92	38.6	0.2
3	R1	Italian	Pasta & vegetable	E	On	H	43	0.13	34.5	0.2
4	R1	Indian	Onion & tomato	E	On	H	38	0.99	36.5	0.3
5	R1	Chinese	Chicken, shrimp & vegetable	E	On	H	38	1.99	230.9	0.8
6	R1	Indian	Chicken & rice	E	On	H	39	1.27	143.7	0.5
7	S1	American	Fried chicken	E	On	M	27	0.30	20.4	0.2
8	S1	American	Fried chicken	E	On	H	11	1.15	78.3	0.3
9	A1	American	Fried chicken	E	Off	M	28	0.35	10.0	0.1
10	A1	American	Fried chicken	E	Off	H	12	1.65	22.2	0.3
11	A2	American	Fried chicken	G	On	M	23	1.73	18.8	0.2
12	A2	American	Fried chicken	G	On	H	14	4.62	98.1	0.5
13	A2	American	Fried chicken	G	Off	M	26	2.65	12.4	0.3
14	A2	American	Fried chicken	G	Off	H	12	6.04	63.7	0.3

Abbreviations: R1, residence 1; S1, student dorm; A1, apartment 1; A2, apartment 2; E, electric stove; G, gas stove; H, High; M, Medium.
